# Effects of Combined Pectoserratus and Pecto-Intercostal Fascial Plane Blocks for Cardiac Surgery via Median Sternotomy: A Randomized Controlled Trial

**DOI:** 10.3390/jcm15134946

**Published:** 2026-06-25

**Authors:** Bosung Kim, Yeong-Gwan Jeon, Jung Hyun So, Soonchang Hong, Ji-Hyoung Park

**Affiliations:** 1 Department of Anesthesiology and Pain Medicine, Wonju College of Medicine, Yonsei University, Wonju 26426, Republic of Korea; lagozzang@yonsei.ac.kr (B.K.); ygjeon@yonsei.ac.kr (Y.-G.J.); ndiesjh@nate.com (J.H.S.); 2 Department of Cardiovascular Surgery, Wonju College of Medicine, Yonsei University, Wonju 26426, Republic of Korea; hongsc93@yonsei.ac.kr

**Keywords:** cardiac surgery, median sternotomy, pectoserratus plane block, pecto-intercostal fascial plane block, visual analog scale, postoperative, pain, QoR-15, opioid consumption

## Abstract

**Background/Objectives**: Ultrasound-guided fascial plane blocks have emerged as opioid-sparing analgesic strategies for cardiac surgery; however, evidence regarding combined block techniques remains limited. This randomized controlled trial evaluated the analgesic efficacy of combined pectoserratus plane block (PSPB) and pecto-intercostal fascial plane block (PIFB) in patients undergoing cardiac surgery via median sternotomy. **Methods**: Sixty-two adult patients undergoing cardiac surgery via median sternotomy were randomized to either a block group receiving bilateral PSPB and PIFB after anesthetic induction or a control group receiving conventional analgesia alone. The primary outcome was postoperative visual analog scale (VAS) pain score at 6, 12, 24, and 48 h after surgery. Secondary outcomes included Korean version of Quality of Recovery-15 (QoR-15K) scores, total opioid consumption, rescue analgesic dose, time to first rescue analgesia, extubation time, intensive care unit (ICU) stay, hospital stay, and the incidence of postoperative nausea and vomiting. **Results**: Fifty-four patients were included in the final analysis. Postoperative VAS scores did not differ significantly between groups after Bonferroni correction for repeated measurements. No significant overall between-group effect was observed in repeated-measures ANOVA. ICU stay was statistically shorter in the block group, although the absolute difference was small and of uncertain clinical relevance. No significant differences were observed in the remaining secondary outcomes. **Conclusions**: Combined PSPB and PIFB did not reduce postoperative pain or improve recovery outcomes after cardiac surgery via median sternotomy. Early postoperative pain scores were numerically higher in the block group, although these differences were not statistically significant after correction for multiple comparisons. The incremental analgesic benefit of combined fascial plane blocks may therefore be limited in this clinical setting.

## 1. Introduction

Median sternotomy remains the standard surgical approach for most cardiac procedures, including coronary artery bypass grafting and valvular surgery. However, extensive tissue dissection, sternal retraction, and manipulation of the thoracic structures frequently result in moderate-to-severe postoperative pain arising from the sternum, ribs, intercostal muscles, and surrounding soft tissues [1]. Adequate postoperative analgesia is therefore essential not only for patient comfort but also for preservation of respiratory function and enhanced postoperative recovery. Traditionally, postoperative pain management after cardiac surgery has relied heavily on systemic opioids. However, high-dose opioid administration is associated with adverse effects such as nausea, vomiting, delayed recovery, and opioid-induced hyperalgesia. These limitations have increased interest in opioid-sparing analgesic strategies within enhanced recovery protocols for cardiac surgery [2].

Regional analgesia has emerged as an important component of multimodal analgesia aimed at reducing perioperative opioid consumption and improving postoperative recovery. In addition to attenuating acute postoperative pain, regional analgesia may reduce central sensitization associated with surgical trauma and opioid exposure, thereby potentially lowering the risk of persistent postoperative pain [3]. Thoracic epidural and paravertebral blocks have demonstrated effective analgesia in cardiac surgery. However, their use may be limited by the risk of serious complications, including neuraxial hematoma or nerve injury, particularly in patients receiving perioperative antithrombotic therapy [4,5].

Recently, ultrasound-guided fascial plane blocks have emerged as safer alternatives for chest wall analgesia because they provide effective analgesia with a lower risk of severe complications [6,7]. Among these techniques, the pectoserratus plane block (PSPB) and the pecto-intercostal fascial plane block (PIFB) have been developed to target the intercostal nerves responsible for anterior chest wall pain following median sternotomy [8,9]. PSPB provides analgesia to the lateral chest wall through spread of local anesthetic to the third to sixth intercostal nerves as well as the long thoracic and intercostobrachial nerves, and its effectiveness in thoracic and cardiac surgery has been reported [10,11]. However, PSPB may not adequately block the anterior cutaneous branches of the intercostal nerves, which are major contributors to pain following median sternotomy [7]. In contrast, PIFB targets the anterior branches of the intercostal nerves corresponding to thoracic dermatomes T2–T6 and has been shown to reduce postoperative pain scores, opioid consumption, and extubation time after cardiac surgery [8,12].

Recent systematic reviews and meta-analyses have suggested that PSPB and PIFB may reduce postoperative pain or opioid consumption after cardiac surgery via median sternotomy. In addition, emerging evidence suggests that both techniques may provide opioid-sparing benefits in this setting. However, most available studies have evaluated parasternal blocks as isolated techniques, and evidence regarding combined fascial plane block strategies designed to cover both lateral and anterior chest wall innervation remains limited [12,13,14]. Considering the complementary anatomical coverage of PSPB and PIFB, the simultaneous use of these two blocks may theoretically provide broader analgesia of the anterior chest wall following median sternotomy. Therefore, this prospective randomized controlled trial was conducted to evaluate the analgesic efficacy of combined PSPB and PIFB in patients undergoing cardiac surgery via median sternotomy. We hypothesized that the combined use of PSPB and PIFB would reduce postoperative pain intensity and improve recovery compared with conventional opioid-based analgesia.

## 2. Materials and Methods

### 2.1. Study Design and Ethical Approval

This prospective, single-center, randomized controlled trial was conducted at Yonsei University Wonju Severance Christian Hospital. The study protocol was initially approved by the Institutional Review Board of Yonsei University in Wonju on 23 July 2025. After a protocol modification, final IRB approval was obtained on 16 September 2025 (IRB No. CR325045). The trial was prospectively registered with the Clinical Research Information Service on 12 August 2025 (CRIS; KCT0010884), before enrollment of the first participant. The first participant was enrolled on 23 September 2025. Written informed consent was obtained from all participants prior to enrollment. Patients were screened, randomized, and analyzed according to CONSORT guidelines.

### 2.2. Participants

We enrolled 62 adult patients (aged ≥ 20 years, body weight ≥ 50 kg) diagnosed with coronary artery disease or valvular heart disease who were scheduled to undergo coronary artery bypass grafting (CABG), off-pump CABG, or cardiac valve surgery via median sternotomy at Wonju Severance Christian Hospital between September 2025 and April 2026. All surgeries were performed by a single cardiovascular surgeon (S.H.).

Exclusion criteria included contraindications to regional blocks, such as coagulopathy (INR > 1.3 or PTT > 43 s without anticoagulant therapy), recent antiplatelet use within 48 h, dual antiplatelet therapy, infection at the injection site, allergy to local anesthetics, or patient refusal. Additional exclusion criteria included the requirement for postoperative therapeutic anticoagulation, severe comorbidities deemed inappropriate by the attending anesthesiologist (e.g., sepsis, empyema, or anatomical abnormalities), emergency surgery, participation in another clinical trial affecting outcomes, and inability to provide informed consent due to illiteracy, cognitive impairment, or language barriers.

### 2.3. Randomization and Blinding

Patients were randomly assigned in a 1:1 ratio to either the block group (PSPB + PIFB) or the control group using a computer-generated random sequence created with Microsoft Excel. Allocation concealment was ensured using sequentially numbered, opaque, sealed envelopes. After induction of anesthesia, the envelope was opened by a designated investigator, and the assigned intervention was performed accordingly. Participants and outcome assessors were blinded to group allocation, whereas the anesthesiologist performing the block procedures was not blinded. Participants were under general anesthesia during block placement and were therefore unaware of group allocation. Postoperative outcome assessors, ICU clinicians, ward clinicians, and attending physicians responsible for postoperative analgesic decisions remained blinded to group allocation throughout the postoperative assessment period. No sham block was performed in the control group.

### 2.4. Perioperative Management

All patients received balanced general anesthesia. Anesthesia was induced using midazolam (0.05 mg/kg), sufentanil (1–2 μg/kg), and rocuronium (0.8 mg/kg). Sevoflurane was used for inhalational induction and maintenance. Anesthesia was maintained with continuous infusion of sufentanil and rocuronium, along with sevoflurane inhalation. After endotracheal intubation, patients were mechanically ventilated using volume-controlled ventilation with a tidal volume of 8 mL/kg. Standard monitoring included electrocardiography (lead II and V) and invasive arterial blood pressure via the right radial artery, with additional femoral arterial monitoring in patients undergoing cardiopulmonary bypass, pulse oximetry, and near-infrared spectroscopy for cerebral oxygenation. Pulmonary artery catheterization (Swan-Ganz^®^ catheter; Edwards Lifesciences LLC, Irvine, CA, USA) was performed to monitor pulmonary artery pressure, central venous pressure, cardiac output, cardiac index, and mixed venous oxygen saturation. Transesophageal echocardiography was used to assess cardiac function intraoperatively. Anesthetic depth was monitored using SedLine electroencephalography (Masimo, Irvine, CA, USA), and sevoflurane concentration was adjusted accordingly. The sufentanil infusion rate was titrated based on overall hemodynamic parameters at the discretion of the attending anesthesiologist. Intraoperative anesthetic management was standardized and applied identically in both groups. The attending anesthesiologist adjusted the sevoflurane concentration and sufentanil infusion according to the institutional anesthesia protocol, based on hemodynamic parameters and anesthetic depth monitoring. Patients were transferred to the intensive care unit (ICU) in an intubated state, and extubation was performed postoperatively according to institutional protocols. After ICU admission, intravenous patient-controlled analgesia (PCA) with fentanyl was initiated for postoperative pain management. The PCA device contained a total volume of 100 mL, with the fentanyl concentration adjusted to 18.5 μg/kg, delivered as a continuous infusion at 2 mL/h with a bolus dose of 1 mL and a 15 min lockout interval. Postoperative analgesic consumption was recorded as morphine milligram equivalents (MME). Postoperative analgesic management was standardized according to a single attending cardiovascular surgeon-directed institutional protocol and order set in both the ICU and surgical ward. All patients received the same fentanyl-based PCA regimen after ICU admission, and rescue analgesics were administered when patients reported moderate pain or greater, defined as a VAS score ≥ 40 mm on the 0–100 mm scale. ICU and ward physicians, who remained blinded to group allocation, assessed postoperative pain and prescribed rescue analgesics according to this protocol, and assigned nurses administered the medications accordingly. Intravenous tramadol (50 mg) or oral Ultracet^®^ (tramadol 75 mg/acetaminophen 650 mg; Janssen Korea Ltd., Seoul, Republic of Korea) was used as the rescue analgesic according to the standardized order set. NSAIDs were not included in the routine postoperative analgesic protocol; one patient received an NSAID for fever management rather than analgesia. Patients were withdrawn from the study if the surgical plan changed or if block-related complications such as local anesthetic systemic toxicity occurred.

### 2.5. Interventions

#### 2.5.1. Block Group

After induction of anesthesia, patients in the block group received bilateral PSPB and PIFB under ultrasound guidance using an in-plane technique with a 21-gauge echogenic needle (Echoplex^®^; Vygon, France). For PSPB, the patient was placed in the supine position and the injection site was sterilized. The ultrasound transducer was positioned below the clavicle along the midclavicular line to identify the subclavian vessels, then moved inferolaterally to the level of the third rib or third intercostal space. The probe was slightly rotated medially to visualize the pectoralis major, pectoralis minor, and serratus anterior muscles. The needle was inserted using an in-plane approach from cephalad to caudad and positioned between the pectoralis minor and serratus anterior muscles. After negative aspiration, 20 mL of 0.25% ropivacaine mixed with epinephrine (1:200,000) was injected on each side. For PIFB, the sternal angle was palpated to identify the second intercostal space. The ultrasound transducer was placed 2–3 cm lateral to the sternum, parallel to it, to visualize the pectoralis major, intercostal muscles, internal thoracic vessels, and transversus thoracis muscle. The needle was inserted into the pecto-intercostal fascial plane at the second and fourth intercostal spaces, and 5 mL of 0.25% ropivacaine with epinephrine (1:200,000) was injected at each site bilaterally. The total dose of ropivacaine administered for the combined blocks was limited to 3 mg/kg (maximum 150 mg). The PSPB and PIFB techniques were performed as previously described in the literature [11,15,16]. During each block procedure, correct needle tip position and apparent fascial plane hydrodissection were confirmed under real-time ultrasound guidance.

#### 2.5.2. Control Group

Patients in the control group received conventional analgesia without regional blocks. All other perioperative anesthetic and postoperative analgesic protocols were identical to those used in the block group.

### 2.6. Outcomes

The primary outcome was postoperative pain intensity measured using a 0–100 mm visual analog scale (VAS) at 6, 12, 24, and 48 h after surgery, where 0 represents no pain, and 100 represents the worst imaginable pain. The highest VAS score reported at each time point was used for quantitative analysis.

Secondary outcomes included the validated Korean version of the Quality of Recovery-15 (QoR-15K), a questionnaire designed to assess postoperative recovery after anesthesia, measured preoperatively and at 24 and 48 h postoperatively; this instrument also includes items that allow additional evaluation of postoperative pain and symptoms such as nausea and vomiting [17,18,19]. Additional secondary outcomes included total opioid consumption (expressed as morphine milligram equivalents [MME], using conversion factors based on the method described by Nielsen et al. [20]); total rescue analgesic dose; time to first rescue analgesic administration; time from the end of surgery to extubation; length of ICU stay; length of hospital stay; and the incidence of postoperative nausea and vomiting using the QoR-15K nausea/vomiting questionnaire.

Additional exploratory outcomes included perioperative hemodynamic variables to assess potential block-related adverse effects.

### 2.7. Sample Size Calculation

The sample size was calculated using G*Power 3.1.9.7 (Heinrich Heine University Düsseldorf, Düsseldorf, Germany), based on a previous study that assessed postoperative pain using a 100 mm VAS but reported the values after conversion to a 0–10 scale [10]. In that study, the mean postoperative pain score in patients receiving opioid-based intravenous PCA after cardiac surgery was 4.1 ± 1.07 on the converted 0–10 scale. A reduction of 1 point on this converted 0–10 scale was considered clinically significant, corresponding to a 10 mm difference on the 0–100 mm VAS used in the present study, and a 10 mm change on the 100 mm postoperative pain VAS has been reported to represent the minimal clinically important difference after surgery [21]. Because this conversion represents a linear rescaling of the same pain scale, the assumed standardized effect size was preserved. With a type I error of 0.05 and a power of 90%, 26 patients per group were required. Considering a 15% dropout rate, 31 patients were enrolled in each group, for a total of 62 patients.

### 2.8. Statistical Analysis

Statistical analyses were performed using SPSS version 29 (IBM Corp., Armonk, NY, USA). Normality of continuous variables was assessed using the Shapiro–Wilk test. Continuous variables were analyzed using the independent *t*-test when normally distributed and the Mann–Whitney *U* test when not normally distributed. Categorical variables were analyzed using the chi-square test. For repeatedly measured variables, post hoc comparisons were adjusted using the Bonferroni correction. In addition, repeated-measures ANOVA was performed as a sensitivity analysis to account for within-patient correlation across repeated VAS measurements. Time was included as the within-subject factor, and group allocation was included as the between-subject factor. Mauchly’s test was used to assess the sphericity assumption, and Greenhouse–Geisser correction was applied when sphericity was violated. For graphical presentation of treatment effects, unadjusted regression coefficients and corresponding 95% confidence intervals were estimated using separate univariable linear regression models for each postoperative VAS time point, including VAS at 6 h despite its non-normal distribution. Variable-adjusted linear regression analyses were performed for primary outcomes to estimate the treatment effect before and after adjustment for this imbalance. As an exploratory sensitivity analysis, multivariable linear regression was performed for early postoperative VAS scores at 6 and 12 h to assess whether the observed between-group differences persisted after adjustment for potential confounding factors. For the multivariable regression models, residual normality was assessed by visual inspection of Q-Q plots, and multicollinearity was assessed using variance inflation factors. Covariates were selected based on baseline imbalance and clinical relevance to postoperative pain after cardiac surgery. These analyses were considered exploratory because of the limited sample size and were not intended to provide confirmatory causal estimates. The primary analysis was conducted using a per-protocol complete-case population. Missing outcome data were not imputed. A two-sided *p*-value < 0.05 was considered statistically significant.

### 2.9. Safety Assessment

Patients were monitored for potential complications related to regional blocks, including pneumothorax, hematoma, and local anesthetic systemic toxicity (LAST). Ultrasound guidance was used to minimize procedural risks, and epinephrine was added to detect inadvertent intravascular injection. The total dose of ropivacaine was maintained within recommended safety limits. Lipid emulsion was readily available for the treatment of suspected local anesthetic systemic toxicity [22]. Because the blocks were performed after induction of general anesthesia, neurologic symptoms suggestive of local anesthetic systemic toxicity could not be directly assessed; therefore, heart rate and mean arterial pressure were monitored and recorded after block performance as part of surveillance for possible systemic toxicity. This safety consideration is supported by reports indicating that LAST may occur during fascial plane blocks and that a substantial proportion of reported pediatric LAST cases occurred under general anesthesia [23,24].

## 3. Results

**Figure 1:**
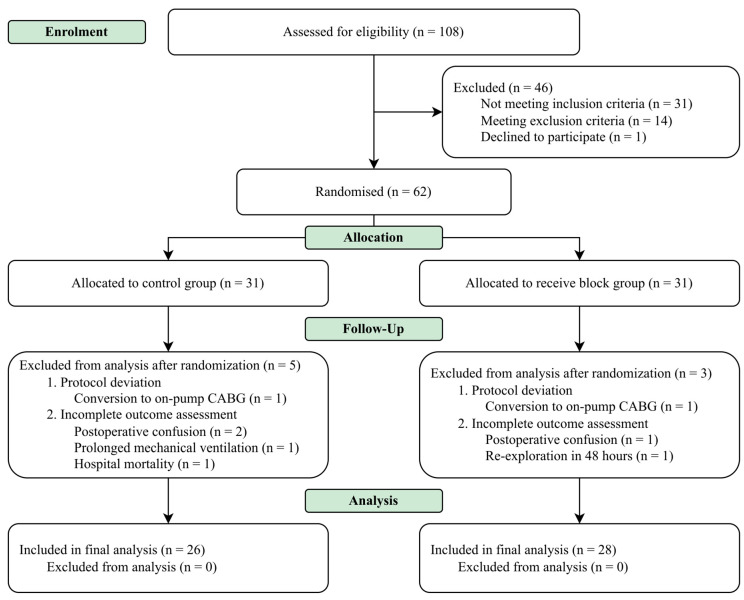
CONSORT flow diagram. Initially, 108 patients were assessed for eligibility. Of these, 46 were excluded, including 31 who did not meet the inclusion criteria, 14 who met the exclusion criteria, and 1 who declined to participate.

A total of 108 patients were assessed for eligibility. Of these, 46 patients were excluded before randomization: 31 did not meet the inclusion criteria, 14 met the exclusion criteria, and 1 declined to participate. The remaining 62 patients were randomized to either the block group (*n* = 31) or the control group (*n* = 31). Among them, 2 underwent unplanned conversion to on-pump CABG intraoperatively, 3 had incomplete outcome assessment due to confusion, 1 required prolonged mechanical ventilation, 1 underwent re-exploration within 48 h postoperatively, and 1 died in the immediate postoperative period because of massive hemorrhage caused by surgical-site rupture before postoperative VAS assessment could be performed. The death was not considered related to the study intervention. These patients were excluded from the per-protocol complete-case analysis. Therefore, 54 patients were included in the final analysis (Figure 1). Among these 54 patients, no missing data were present for variables included in the baseline, primary outcome, secondary outcome, or regression analyses (0/54, 0.0% for each variable). Baseline patient characteristics and surgical data are presented in Table 1. There were no significant differences in baseline demographic characteristics between the two groups, except for diabetes mellitus.

**Figure 2:**
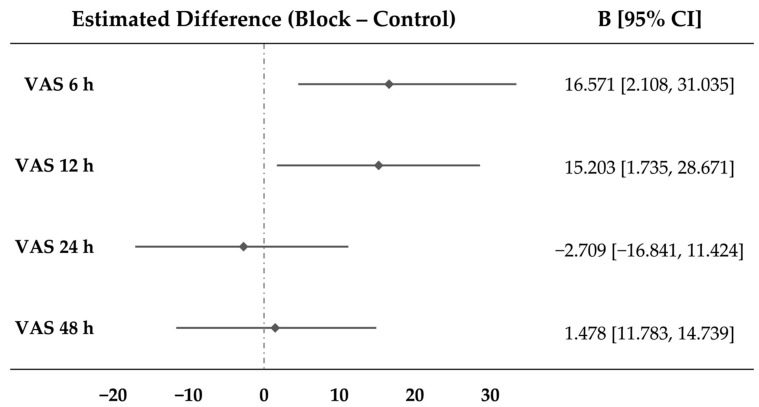
Unadjusted Regression coefficients for VAS scores at each time point. Point estimates represent regression coefficients for the difference in postoperative VAS scores between the block and control groups, calculated as block group minus control group. Regression coefficients obtained from separate univariable linear regression models with group allocation as the sole predictor. Horizontal bars indicate 95% confidence intervals. The vertical dashed line represents no between-group difference. Positive values indicate higher VAS scores in the block group, whereas negative values indicate lower VAS scores in the block group. B values represent regression coefficients. VAS, visual analog scale; CI, confidence interval.

In unadjusted comparisons, postoperative VAS scores at 6 and 12 h were higher in the block group (6 h: 70.00 [60.00–81.50] vs. 45.00 [30.00–80.00] *p* = 0.034; 12 h: 66.86 ± 24.30 vs. 51.65 ± 25.01; *p* = 0.028). However, these differences did not remain statistically significant after Bonferroni correction for repeated measurements (adjusted *p* = 0.136 and 0.112, respectively). The incidence of severe pain, defined as a VAS score ≥70 mm, was also higher in the block group at 12 h in the unadjusted comparison (57.1% vs. 26.9%; *p* = 0.025), but not after correction (adjusted *p* = 0.050). There were no significant differences in VAS scores at 24 and 48 h postoperatively between the two groups. The magnitude and precision of the between-group differences in postoperative VAS scores are illustrated in Figure 2. In the repeated-measures ANOVA performed as a sensitivity analysis, Mauchly’s test indicated that the sphericity assumption was violated (*p* < 0.001); therefore, Greenhouse–Geisser-corrected results were used. There was a significant main effect of time on postoperative VAS scores (F = 13.629, *p* < 0.001) and a significant time-by-group interaction (F = 3.289, *p* = 0.038). The overall between-group effect was not statistically significant (F = 2.231, *p* = 0.141). These findings indicate that the postoperative pain trajectory differed between groups, mainly reflecting higher early VAS scores in the block group and a subsequent reduction in the between-group difference over time. This analysis was consistent with the time point-specific comparisons after Bonferroni correction and did not alter the overall interpretation of the primary outcome.

QoR-15K scores at 24 and 48 h postoperatively did not differ significantly between the groups. In the QoR-15K nausea/vomiting domain at 48 h postoperatively, the block group showed a lower median score than the control group (9.00 [6.00–10.00] vs. 10.00 [10.00–10.00]; *p* = 0.005). Because higher QoR-15K scores indicate better recovery, this difference should not be interpreted as an improvement in the block group. However, median scores remained high in both groups. The length of ICU stay was shorter in the block group, although the absolute difference in median values was small (24.00 vs. 26.05 h, *p =* 0.045). No significant differences were observed between the groups in time to first rescue analgesic administration, rescue analgesic consumption, total analgesic consumption, proportion of patients requiring rescue analgesia, length of hospital stay, or time from the end of surgery to extubation (Table 2). In addition, no significant differences were observed in any of the five QoR-15K domains assessed preoperatively and at 24 and 48 h postoperatively, or in the changes from baseline QoR-15K scores (Table 3). No block-related adverse events were reported among the study participants.

Because the prevalence of diabetes mellitus differed significantly between the groups at baseline, additional regression analyses were performed to assess whether this imbalance influenced the estimated treatment effects for the primary outcomes. In diabetes-adjusted linear regression models, the estimated between-group differences in postoperative VAS scores were generally consistent with the unadjusted estimates, and group allocation was not significantly associated with VAS scores at any postoperative time point (Table A1). Diabetes mellitus was not independently associated with postoperative VAS scores at either 6 h (B = 4.257, *p =* 0.585) or 12 h (B = 0.768, *p =* 0.912) (Table A2).

## 4. Discussion

### 4.1. Interpretation

Pectoral fascial (PECS) II block, including PSPB, was first introduced in 2012 as a modification of PECS I block [25]. It is known to provide coverage of lateral cutaneous branches of the third to sixth intercostal nerves and the long thoracic nerve [11]. One study demonstrated that PECS I and II blocks may contribute to postoperative analgesia in cardiac surgery [26]. However, because PECS II block spares anterior branches of intercostal nerves, it may not provide sufficient analgesic coverage for pain associated with cardiac surgery [6,27]. PIFB was subsequently introduced in 2014 as an adjunct to PECS blocks [8]. Meanwhile, another parasternal block technique, transverse thoracic muscle plane block (TTMPB), was introduced in 2015 and was reported to provide clinically meaningful analgesia for breast surgery when combined with PECS II block [28]. Subsequent studies also suggested that TTMPB could provide analgesic coverage applicable to cardiac surgery [29]. However, the transverse thoracic muscle is a very thin structure that is difficult to identify using ultrasound, making TTMPB technically challenging to perform [30]. Therefore, PIFB was proposed as an easier and safer alternative to TTMPB [31]. A study demonstrated that a double-injection technique at the second and fourth costal cartilages provided more reliable coverage of T2–T6 dermatomes of the anteromedial thoracic chest wall than a single-injection approach, with coverage comparable to that of TTMPB [16]. Therefore, combining PSPB and PIFB was expected to provide broader and more comprehensive analgesic coverage of the anterior and lateral thoracic wall in patients undergoing median sternotomy.

Although recent systematic reviews and meta-analyses have generally supported the analgesic or opioid-sparing potential of fascial plane blocks, whether these findings can be extrapolated to a combined lateral and parasternal block strategy remains uncertain. In the present trial, the combined use of PSPB and PIFB did not improve postoperative pain outcomes. Contrary to our original hypothesis, early postoperative VAS scores at 6 and 12 h and the incidence of severe pain at 12 h showed an unfavorable numerical direction in the block group in unadjusted comparisons. These time point-specific differences did not remain statistically significant after Bonferroni correction, and repeated-measures ANOVA showed no statistically significant overall between-group effect despite a difference in postoperative VAS trajectories. No significant differences were observed in other recovery-related outcomes, including QoR-15K scores and opioid consumption. The statistically significant differences observed in secondary outcomes should also be interpreted cautiously. Although length of ICU stay was shorter in the block group, the absolute difference was only approximately 2.05 h, which is unlikely to represent a clinically meaningful improvement in postoperative recovery. This small difference may also reflect institutional workflow or discharge timing rather than a clinically meaningful recovery benefit. Similarly, the difference in the QoR-15K nausea/vomiting item at 48 h should not be overinterpreted. Because higher QoR-15K scores indicate better recovery, the lower nausea/vomiting score observed in the block group does not support a beneficial effect of the combined block strategy. Moreover, this finding occurred without reductions in total opioid consumption or rescue analgesic requirements. Therefore, our findings should not be interpreted as supporting the analgesic efficacy of this combined block strategy in patients undergoing cardiac surgery via median sternotomy. Several hypotheses may explain these unexpected findings and the lack of clinically meaningful benefit despite previously reported favorable findings for individual fascial plane blocks.

The analgesic efficacy of fascial plane blocks in cardiac surgery via median sternotomy may be less pronounced than previously anticipated. Postoperative pain after cardiac surgery is multifactorial, arising not only from musculoskeletal injury but also from visceral and mediastinal pain, mechanical injury caused by sternal retractors, neuropathic pain associated with internal mammary artery harvesting, and irritation from chest tubes [32]. Although PSPB and PIFB were combined to provide complementary analgesic coverage of the lateral and parasternal anterior chest wall, their anatomical targets are mainly related to chest wall somatic innervation and may not fully address deep sternal pain, visceral or mediastinal pain, chest tube-related pain, or neuropathic pain associated with internal mammary artery harvesting. In addition, a recent study reported that continuous erector spinae plane block (ESPB) using a catheter technique in cardiac surgery did not provide superior analgesia or opioid-sparing effects compared with intravenous lidocaine infusion [33]. Furthermore, plasma lidocaine concentrations exceeded the generally accepted therapeutic range in some patients who received ESPB. These findings raise the possibility that the apparent analgesic effects of ESPB in cardiac surgery may be attributable, at least in part, to systemic local anesthetic absorption rather than solely to a regional analgesic mechanism. Similarly, PSPB and PIFB may not consistently provide reliable dermatomal sensory blockade.

Although opioid-sparing strategies are increasingly emphasized because high-dose opioid techniques are associated with delayed recovery, prolonged mechanical ventilation, increased ICU stay, and opioid-induced hyperalgesia, reducing opioid use in cardiac surgery remains challenging because of the cardioprotective effects of opioids and the unique hemodynamic and intraoperative characteristics of cardiac surgery [34]. Therefore, the relatively high systemic opioid exposure associated with cardiac anesthesia and postoperative opioid-based PCA may have attenuated the incremental analgesic benefit of PSPB and PIFB. Consequently, potential block-related reductions in postoperative pain and opioid consumption may have been difficult to distinguish from the effects of systemic opioid administration.

Rebound pain may be considered one possible hypothesis for the unexpected early postoperative pain pattern; however, this interpretation remains speculative. Although rebound pain has not been uniformly defined, it has been described as a transient state of hyperalgesia occurring approximately 8–24 h after block administration and lasting for 2–6 h, distinct from persistent postsurgical pain (PPSP) [35,36]. The precise mechanism remains unclear; however, transient hyperalgesia following resolution of peripheral nerve block (PNB) has been proposed as a possible mechanism. Reported risk factors for rebound pain include moderate-to-severe postoperative pain and single-injection PNB techniques. Strategies proposed to reduce rebound pain include continuous catheter techniques, prolongation of block duration using adjuncts such as dexamethasone, and systemic multimodal analgesia [37]. In the present trial, regional blocks were performed immediately after anesthetic induction rather than at the end of surgery. Therefore, the postoperative 6 and 12 h VAS assessments approximately corresponded to 12 and 18 h after block administration, respectively, which is consistent with the previously described time window for rebound pain. In addition, the use of single-injection PNB techniques without adjuncts to prolong block duration may have further contributed to this phenomenon. Nevertheless, rebound pain has been primarily described following dense sensory PNBs, such as brachial plexus or popliteal sciatic nerve blocks, and evidence regarding rebound pain after fascial plane blocks remains limited. Furthermore, block duration, postoperative sensory recovery, and dermatomal coverage were not assessed in this study. Therefore, rebound pain cannot be confirmed as a mechanism underlying the observed findings and should be interpreted only as a possible explanation.

From a clinical perspective, the present findings do not demonstrate a clear advantage of combining PSPB and PIFB over conventional opioid-based multimodal analgesia. The rationale for this combined strategy was based on the complementary anatomical coverage of PSPB for the lateral chest wall and PIFB for the parasternal anterior chest wall. However, this anatomical rationale did not translate into measurable improvements in postoperative VAS scores, opioid consumption, rescue analgesic requirements, QoR-15K scores, extubation time, or hospital length of stay. Although both blocks are technically feasible under ultrasound guidance, their combined use requires bilateral multi-site injections and a relatively large total volume of local anesthetic compared with simpler single-block or parasternal approaches. Therefore, based on the current data, the practical necessity and additional clinical value of routinely performing combined PSPB and PIFB remain uncertain. Future studies should directly compare this combined strategy with simpler regional analgesic approaches to determine whether any specific patient subgroup may benefit from a more extensive block strategy.

### 4.2. Strengths

Recent enhanced recovery after cardiac surgery (ERACS) protocols increasingly emphasize opioid-sparing multimodal analgesia and have led to growing interest in regional analgesic techniques for cardiac surgery [38]. Although thoracic epidural analgesia has demonstrated favorable analgesic efficacy, concerns regarding epidural hematoma or hemorrhagic complications associated with high-dose perioperative heparinization have promoted the development and application of fascial plane blocks in cardiac surgery [6]. However, studies evaluating combined fascial plane block strategies in cardiac surgery remain scarce. To the best of our knowledge, only a few randomized controlled trials have evaluated combined fascial plane block techniques in this setting, including combined PIFB with rectus sheath block and combined ESPB with superficial parasternal intercostal plane block [39,40,41]. The present study evaluated the efficacy of a previously unstudied combination of PSPB and PIFB in a prospective randomized controlled trial. Furthermore, the inclusion of QoR-15K in addition to pain scores and opioid consumption allowed a more comprehensive assessment of postoperative recovery and patient-centered outcomes [42].

### 4.3. Limitations

This study has several limitations. Because this was a single-center study with a relatively small sample size, the findings may have limited external validity. In addition, the sample size was calculated based on the primary VAS pain outcome and was not powered to detect differences in secondary outcomes, including opioid consumption, QoR-15K scores, length of ICU stay, extubation time, or other postoperative recovery variables. Therefore, findings related to these secondary outcomes should be interpreted as exploratory.

The inclusion of a heterogeneous surgical population, including CABG, valve surgery, and combined CABG with valve surgery, may have introduced variability in postoperative pain characteristics and analgesic requirements. Although all procedures were performed via median sternotomy, they may differ in operative trauma, chest tube burden, internal mammary artery harvesting, and postoperative pain patterns. Differences in pleural chest tube placement and operative duration may also have influenced postoperative pain outcomes. Because of the small sample size, meaningful subgroup analyses according to surgical procedure were not feasible. Therefore, surgical heterogeneity may have influenced pain outcomes and limited the ability to identify procedure-specific effects of the combined block strategy.

Because combined blocks required relatively large volumes of local anesthetic, lower concentrations were used to remain within the recommended safe dose range. Although previous studies have reported no significant difference in block duration between 0.2% and 0.375% ropivacaine concentrations [43], the lower concentration used in this study may nevertheless have attenuated block efficacy or shortened block duration in the setting of moderate-to-severe postoperative pain after cardiac surgery.

Postoperative sensory block assessment was not performed. Because the blocks were administered after anesthetic induction and patients were transferred to the ICU while intubated and sedated, immediate and reliable sensory testing was not feasible. Therefore, the actual block success rate and dermatomal sensory coverage could not be confirmed, and the findings should be interpreted as evaluating the clinical strategy of administering combined PSPB and PIFB after anesthetic induction rather than the analgesic efficacy of confirmed successful blockade.

Although total postoperative opioid consumption was recorded, time-resolved PCA use at each postoperative assessment point and PCA demand/delivery ratios could not be reliably determined because the exact residual volume and device-derived demand data were not available. Therefore, temporal changes in PCA demand or consumption could not be analyzed in relation to VAS scores at each time point.

The primary analysis was performed using a per-protocol complete-case population rather than a strict intention-to-treat population. Eight randomized patients were excluded after allocation because of major protocol deviations, incomplete outcome assessment, or postoperative events that precluded reliable VAS assessment. Although these exclusions were clinically justified, post-randomization exclusion may have introduced attrition bias and reduced internal validity. Therefore, the findings should be interpreted as per-protocol complete-case estimates rather than strict intention-to-treat estimates.

Blinding was incomplete. Patients, postoperative outcome assessors, ICU clinicians, ward clinicians, and attending physicians responsible for postoperative analgesic decisions were blinded to group allocation, and no sham block was performed in the control group. In addition, the anesthesiologist performing the block and providing intraoperative anesthetic management could not be blinded. Although intraoperative anesthetic management was standardized and postoperative rescue analgesics were administered according to a predefined institutional protocol by blinded clinicians, the possibility of performance bias cannot be completely excluded.

Finally, although diabetes-adjusted and exploratory multivariable analyses were performed, residual confounding cannot be excluded. Diabetes mellitus is associated with increased postoperative mortality, greater postoperative opioid requirements, and delayed recovery, including prolonged mechanical ventilation and longer ICU and hospital stays [44,45]. Diabetic neuropathy may also be associated with altered pain characteristics after regional analgesia [46]. In the present trial, detailed information regarding the presence, duration, severity, and treatment status of diabetes-related complications was not assessed. Therefore, the baseline imbalance in diabetes mellitus should be considered when interpreting the observed early postoperative pain outcomes.

## 5. Conclusions

In conclusion, the combined use of PSPB and PIFB did not reduce postoperative pain or improve recovery outcomes after cardiac surgery via median sternotomy. Rather, early postoperative VAS scores and the incidence of severe pain showed an unfavorable numerical direction in the block group, although these findings did not remain statistically significant after correction for multiple comparisons. Given the absence of improvements in opioid consumption, rescue analgesic requirements, QoR-15K scores, extubation time, and hospital length of stay, the present findings do not support a clinically meaningful analgesic advantage of combined PSPB and PIFB over standard analgesic management in this clinical setting. Accordingly, the routine use of this combined fascial plane block strategy cannot be recommended based on the current data. Further studies are needed to determine whether simpler regional analgesic approaches or modified block strategies can provide clinically meaningful benefits for patients undergoing cardiac surgery via median sternotomy.

## Figures and Tables

**Table 1:** Patients’ characteristics and surgery data.

	Control Group (*n* = 26)	Block Group (*n* = 28)	*p*-Value
Age (years)	66.50 ± 8.46 [45, 79]	67.61 ± 8.60 [50, 84]	0.636
Male	23 (88.5)	21 (75)	0.203
Height (cm)	163.78 ± 7.59 [147.0, 182.7]	161.74 ± 7.34 [148.5, 174.0]	0.321
Weight (kg)	67.41 ± 10.28 [52.5, 95.8]	65.04 ± 9.91 [50.2, 88.7]	0.392
Hypertension	20 (76.9)	26 (92.9)	0.100
Diabetes mellitus	11 (42.3)	21 (75)	0.015 *
Prior myocardial infarction	6 (23.1)	5 (17.9)	0.634
Coronary artery occlusive disease	18 (69.2)	20 (71.4)	0.860
Peripheral artery occlusive disease	2 (7.7)	1 (3.6)	0.509
Cerebrovascular accident	5 (19.2)	5 (17.9)	0.897
Chronic kidney disease	3 (11.5)	5 (17.9)	0.514
Dyslipidemia	12 (46.2)	9 (32.1)	0.291
NYHA class			0.698
I	9 (34.6)	8 (28.6)	—
II	12 (46.2)	15 (53.6)	—
III	4 (15.4)	5 (17.9)	—
IV	1 (3.8)	0 (0)	—
EuroSCORE II (%)	1.20 [0.71–1.94]	1.31 [0.97–2.40]	0.250
Ejection fraction (%)	55.00 [38.75–61.25]	59.50 [49.75–65.00]	0.080
Baseline QoR-15K	140.00 [132.75–147.25]	137.50 [124.25–150.00]	0.828
Surgery time (min)	262.88 ± 54.65 [166, 374]	241.21 ± 61.65 [150, 425]	0.179
Anesthesia time (min)	350.00 [295–385]	330 [285–379.50]	0.573
Surgery type			0.322
Valve	8 (30.8)	10 (35.7)	—
CABG	18 (69.2)	16 (57.1)	—
Valve with CABG	0 (0)	2 (7.1)	—
Use of cardiopulmonary bypass	9 (34.6)	13 (46.4)	0.377
Pleural chest tube insertion	13 (50)	12 (42.9)	0.599

Data are presented as mean ± SD [minimum, maximum], median [Q1–Q3], or number (%), as appropriate. NYHA, New York Heart Association; QoR-15K, Korean version of the Quality of Recovery-15; CABG, coronary artery bypass grafting. Valve surgery includes aortic valve replacement, mitral valve repair, mitral valve replacement, and tricuspid annuloplasty; CABG surgery includes on-pump and off-pump coronary artery grafting. * = *p*-value < 0.05.

**Table 2:** Outcome comparisons between the groups.

	Control Group (*n* = 26)	Block Group (*n* = 28)	*p*-Value	Mean Difference (95% CI)
Primary outcomes				
VAS 6 h	45.00 [30.00–80.00]	70.00 [60.00–81.50]	0.034 (0.136)	—
VAS 12 h	51.65 ± 25.01 [0, 100]	66.86 ± 24.30 [10, 100]	0.028 (0.112)	15.20 (1.74, 28.67)
VAS 24 h	50.92 ± 24.23 [0, 91]	48.21 ± 27.28 [0, 90]	0.702 (1.000)	−2.71 (−16.84, 11.42)
VAS 48 h	37.81 ± 24.14 [0, 87]	39.29 ± 24.38 [0, 90]	0.824 (1.000)	1.48 (−11.78, 14.74)
VAS (70–100) 6 h	9 (34.6)	16 (57.1)	0.097 (0.194)	—
VAS (70–100) 12 h	7 (26.9)	16 (57.1)	0.025 (0.050)	—
Secondary outcomes				
QoR-15K Total 24 h	94.00 ± 24.29	94.46 ± 27.46	0.948	0.46 (−13.74, 14.66)
QoR-15K Total 48 h	111.54 ± 20.01	107.86 ± 20.46	0.507	−3.68 (−14.75, 7.38)
QoR-15K N/V questionnaire 24 h	10.00 [9.00–10.00]	10.00 [5.00–10.00]	0.084	—
QoR-15K N/V questionnaire 48 h	10.00 [10.00–10.00]	9.00 [6.00–10.00]	0.005 *	—
Time to 1st rescue analgesics	718.62 ± 388.84 [200, 1635]	787.32 ± 317.21 [225, 1290]	0.479	68.71 (−124.47, 261.88)
Rescue analgesic dose (MME)	49.79 ± 26.04 [0, 120]	43.13 ± 24.03 [0, 90]	0.333	−6.66 (−20.34, 7.01)
Total opioid analgesic dose (MME)	415.27 ± 68.71 [315, 585]	399.38 ± 64.34 [322.5, 570]	0.384	−15.89 (−52.23, 20.44)
Total acetaminophen dose (mg)	3354.81 ± 2677.90 [0, 9550]	2652.68 ± 1922.519 [975, 9550]	0.271	−702.13 (−1968.20, 563.95)
Patients requiring rescue analgesia	25 (96.2)	25 (89.3)	0.336	—
New onset atrial fibrillation	0 (0)	3 (10.7)	0.086	—
Wound infection	0 (0)	0 (0)	1.000	—
Mortality	0 (0)	0 (0)	1.000	—
Length of ICU stay (hour)	26.05 [23.38–27.00]	24.00 [21.63–25.75]	0.045 *	—
Length of hospital stay (day)	10.00 [8.00–12.00]	9.00 [8.00–10.00]	0.118	—
Time to extubation (min)	1047.50 [828.75–1146.25]	947.50 [781.25–1097.50]	0.200	—

Data are presented as mean ± SD [minimum, maximum], median [Q1–Q3], or number (%), as appropriate. The pain intensity was measured using a VAS (0 = no pain, 100 = the worst imaginable pain). Mean differences and 95% confidence intervals were calculated from unadjusted between-group comparisons for continuous variables summarized as mean ± SD, using the block group minus control group direction. *p*-values in parentheses indicate Bonferroni-adjusted *p*-values. Rescue analgesic requirement dose was calculated in MME. Length of ICU stay is presented in hours to facilitate clinical interpretation. VAS, visual analog scale; N/V, nausea/vomiting; MME, morphine milligram equivalent; ICU, intensive care unit. SD, standard deviation; CI, confidence interval; Q1–Q3, first quartile–third quartile. * = *p*-value < 0.05.

**Table 3:** QoR-15K between the groups.

	Control Group (*n* = 26)	Block Group (*n* = 28)	*p*-Value	Mean Difference (95% CI)
Baseline				
Emotional state	33.31 ± 6.34 [21, 40]	32.43 ± 7.67 [14, 40]	0.650	−0.88 (−4.74, 2.98)
Physical comfort	47.58 ± 4.16 [33, 50]	46.64 ± 4.56 [35, 50]	0.436	−0.93 (−3.32, 1.45)
Psychological support	19.77 ± 0.71 [17, 20]	19.39 ± 1.62 [14, 20]	0.270	−0.38 (−1.06, 0.30)
Physical independence	18.19 ± 3.91 [2, 20]	17.25 ± 3.87 [7, 20]	0.377	−0.94 (−3.07, 1.18)
Pain	18.08 ± 4.87 [2, 20]	17.21 ± 5.13 [1, 20]	0.530	−0.86 (−3.60, 1.88)
Total	136.92 ± 13.19 [102, 150]	132.93 ± 19.18 [85, 150]	0.380	−4.00 (−13.05, 5.06)
Postoperative 24 h				
Emotional state	27.77 ± 10.58 [5, 40]	28.54 ± 8.89 [9, 40]	0.774	0.77 (−4.56, 6.09)
Physical comfort	33.92 ± 9.47 [14, 50]	34.04 ± 11.55 [6, 50]	0.969	0.11 (−5.68, 5.91)
Psychological support	17.15 ± 4.14 [7, 20]	16.25 ± 4.21 [6, 20]	0.430	−0.90 (−3.19, 1.38)
Physical independence	4.42 ± 5.59 [0, 20]	4.57 ± 4.95 [0, 18]	0.918	0.15 (−2.73, 3.03)
Pain	10.73 ± 6.10 [0, 20]	11.07 ± 5.84 [0, 20]	0.835	0.34 (−2.92, 3.60)
Total	94.00 ± 24.29 [57, 134]	94.46 ± 27.46 [29, 140]	0.948	0.46 (−13.74, 14.66)
Change from baseline	42.92 ± 23.60 [2, 81]	38.46 ± 27.94 [−4, 112]	0.531	−4.46 (−18.64, 9.72)
Postoperative 48 h				
Emotional state	32.85 ± 4.82 [22, 40]	31.82 ± 6.25 [16, 40]	0.506	−1.03 (−4.09, 2.04)
Physical comfort	40.04 ± 7.24 [24, 50]	37.46 ± 9.03 [14, 50]	0.255	−2.57 (−7.06, 1.92)
Psychological support	18.46 ± 3.28 [7, 20]	17.46 ± 2.84 [8, 20]	0.236	−1.00 (−2.67, 0.67)
Physical independence	6.96 ± 6.17 [0, 19]	7.04 ± 5.43 [0, 18]	0.963	0.07 (−3.10, 3.25)
Pain	13.23 ± 5.23 [4, 20]	14.00 ± 4.55 [5, 20]	0.566	0.77 (−1.91, 3.44)
Total	111.54 ± 20.01 [68, 149]	107.86 ± 20.46 [67, 144]	0.507	−3.68 (−14.75, 7.38)
Change from baseline	25.38 ± 22.33 [−21, 69]	25.07 ± 21.10 [−11, 75]	0.958	−0.31 (−12.17, 11.55)

Data are presented as mean ± SD [minimum, maximum]. Mean differences were calculated as block group minus control group. Higher QoR-15K scores indicate better postoperative recovery. SD, standard deviation; CI, confidence interval.

## Data Availability

The data presented in this study are available upon request from the corresponding author.

## Abbreviations

The following abbreviations are used in this manuscript:

PSPB	Pectoserratus plane block
PIFB	Pecto-intercostal fascial plane block
CABG	Coronary artery bypass grafting
LAST	Local anesthetic systemic toxicity
VAS	Visual analog scale
QoR-15K	Korean version of the Quality of Recovery-15
MME	Morphine milligram equivalent

## Appendix A

**Table A1:** Unadjusted and DM-adjusted treatment effects for postoperative VAS scores.

	Unadjusted		Adjusted	
Outcomes	Mean Difference (95% CI)	*p*-Value	Mean Difference (95% CI)	*p*-Value
VAS 6 h	16.57 (2.11, 31.04)	0.026 *	14.15 (−1.21, 29.50)	0.070
VAS 12 h	15.20 (1.74, 28.67)	0.028 *	15.24 (0.81, 29.66)	0.039 *
VAS 24 h	−2.71 (−16.84, 11.42)	0.702	−2.47 (−17.60, 12.67)	0.745
VAS 48 h	1.48 (−11.78, 14.74)	0.824	1.33 (−12.87, 15.54)	0.851

Data are presented as mean differences with 95% confidence intervals, calculated as block group minus control group. In the adjusted model, the mean difference corresponds to the regression coefficient for group allocation from a linear regression model including group allocation and diabetes mellitus. VAS, visual analog scale; CI, confidence interval. * = *p*-value < 0.05.

**Table A2:** Multivariable linear regression analysis for postoperative VAS scores at 6 and 12 h.

	VAS 6 h			VAS 12 h		
Variable	Regression Coefficient (B)	*p*-Value	VIF	Regression Coefficient (B)	*p*-Value	VIF
PSPB + PIFB	10.213	0.179	1.254	8.764	0.197	1.254
Male	−15.517	0.095	1.116	−12.703	0.125	1.116
Age	−0.622	0.164	1.220	−0.477	0.232	1.220
DM	4.257	0.585	1.295	0.768	0.912	1.295
EuroSCORE II (%)	−3.850	0.011 *	1.292	−3.839	0.005 *	1.292
Baseline QoR-15K	−0.543	0.021 *	1.233	−0.565	0.008 *	1.233
Anesthesia Time	0.025	0.660	1.225	0.045	0.377	1.225
Use of cardiopulmonary bypass	12.724	0.222	2.285	28.334	0.004 *	2.285
Additional pleural chest tube	19.442	0.037 *	1.829	25.090	0.003 *	1.829

Data are presented as regression coefficient (B) and *p*-value from multivariable linear regression analysis. Covariates included group allocation, sex, age, diabetes mellitus, EuroSCORE II, preoperative QoR score, anesthesia time, cardiopulmonary bypass, and pleural chest tube insertion. The adjusted R^2^ values were 0.204 and 0.264 for postoperative VAS scores at 6 and 12 h models, respectively. Residual normality was assessed by visual inspection of Q-Q plots, and collinearity diagnostics demonstrated no significant multicollinearity among predictors (VIF range, 1.116–2.285). VIF, variance inflation factor. * = *p*-value < 0.05.
